# Leydig cell tumor of the testis with azoospermia and elevated delta4 androstenedione: case report

**DOI:** 10.1186/s12610-016-0041-8

**Published:** 2016-11-08

**Authors:** J. Prasivoravong, A-L. Barbotin, A. Derveaux, C. Leroy, X. Leroy, P. Puech, V. Mitchell, F. Marcelli, J-M. Rigot

**Affiliations:** 1 Department of Andrology, Lille University Hospital, Lille, France; 2 Biology of Reproduction Unit, Lille University Hospital, Lille, France; 3 Department of Pathology, Lille University Hospital, Lille, France; 4 Department of Radiology, Lille University Hospital, Lille, France; 5 EA4308 Gametogenesis and Gamete Quality, University of Lille, Lille, France; 6 Department of Andrology, CHRU Lille, Hôpital Calmette, Boulevard du Professeur Leclercq, 59037 Lille Cedex, France

**Keywords:** Azoospermia, Infertility, Hormone secreting testicular tumor, Leydig cell tumor, delta4 androstenedione, TESE, Azoospermie, Infertilité, Tumeur testiculaire à sécrétion endocrine, Tumeur à cellules de Leydig, delta4androstenedione, TESE

## Abstract

**Background:**

Secreting interstitial cell (Leydig cell) tumors are rare. In adults, the clinical picture and steroid levels are variable.

**Case presentation:**

This paper presents a case of left testicular tumor, showing azoospermia with normal serum level of total testosterone, collapsed FSH and LH, and high delta4 androstenedione. Histopathological investigation revealed a Leydig cell tumor. TESE allowed spermatozoa extraction and freezing. Testicular histology found hypospermatogenesis and germ-cell aplasia with interstitial fibrosis. Surgical resection of the tumor resulted in normalization of gonadotropins and fall in serum delta4 androstenedione to subnormal levels in the postoperative period confirming that the tumor was secreting delta4 androstenedione. It was hypothesized that high delta4 androstenedione resulted in intra tumoral 17 β-HSD overtaken by delta4 androstenedione or that 17 β-HSD activity in the tumor was different from that of normal Leydig cells. Three months after surgery sperm analysis found a complete recovery of spermatogenesis. A spontaneous pregnancy occurred 3 months after surgery and a girl was born.

**Conclusions:**

In this case, the diagnosis of testicular Leydig cell tumor secreting delta4 androstenedione was made in a context of azoospermia.

## Résumé

**Introduction:**

Les tumeurs testiculaires interstitielles (ou tumeurs testiculaires à cellules de Leydig) à expression endocrine sont rares. Chez l’adulte le tableau clinique et le bilan hormonal sont variables.

**Présentation du cas:**

Cet article présente le cas d’une tumeur testiculaire gauche dans un contexte d’azoospermie. Le bilan hormonal montre des gonadotrophines effondrées, une testostéronémie normale et une delta4 androstenedione augmentée. L’examen anatomopathologique a mis en évidence une tumeur à cellule de Leydig. La TESE a permis l’extraction et la congélation de spermatozoïdes. L’histologie a retrouvé un aspect mixte d’hypospermatogenèse diminuée incomplète et d’aplasie. Dans les suites de l’orchidectomie partielle gauche les taux de gonadotrophines se sont normalisés ainsi que le taux de delta4 androstenedione. L’hypothèse physiopathologique est que l’augmentation de la delta4 androstenedione résulte de la sursaturation de la 17 β-HSD intra-tumoral ou que l’activité de la 17 β-HSD intra-tumoral est différente de celle dans les cellules de Leydig normales. Trois mois après la chirurgie, le spermogramme a montré une normalisation des paramètres spermatiques et une grossesse spontanée est survenue permettant la naissance d’une petite fille.

**Conclusion:**

Dans ce cas clinique, le diagnostic de tumeur testiculaire à cellule de Leydig sécrétant de la delta4 androstenedione a été fait dans un contexte d’azoospermie.

## Background

Testicular neoplasms represent 1–1.5 % of all tumors in men. Those derived from the interstitial cells of Leydig are rare, constituting 1 % of testicular tumors. Hormone secreting interstitial cell tumors are more unusual than non-secreting interstitial tumors. In young males, the tumor is usually associated with precocious puberty [1], whereas in adults, the clinical picture and steroid levels are variable [2–20]. This paper reports a case of testicular Leydig cell (interstitial cell) tumor secreting delta4 androstenedione in a patient with azoospermia.

## Case presentation

A 44 year old man was referred to our clinic for secondary infertility; he had no medical or surgical history or medical treatment. He was a butcher and married.

In 2009, he consulted another medical center for primary infertility. His medical history included no testicular trauma, no inguino scrotal surgery, no cryptorchidism, no professional exposure, no tobacco, no alcohol, or drug consumption. Semen analysis is summerized in Table 1.

**Table 1:** Semen analyses

	2009	Under 2014	Under 3 months
Volume (mL)	4.2	4.3	3.5
Spermatozoa (10^6^/ejaculate)	0.78	0	259
Progressive motility (%)	19	0	60
Typical spermatozoa (%)	NF	0	33

*NF* not feasible

Serum level of total testosterone was 5.03 mg/L, FSH 1.0 IU/L. Spermoculture found ureaplasma urealyticum.

Scrotal ultrasonography (Table 2) revealed:

**Table 2:** Scrotal ultrasonography

	2009	2014 before surgery	3 months after surgery
Right testis	Normal in size and echotexture, without focal lesion; normal epididymides; no varicocele; normal vasculature at Doppler examination	Normal echotexture Volume 8.5 mL	Normal echotexture Volume 15.7 mL
Left testis	Presence of a 30 × 16 mm polylobulated intratesticular mass, with low echogenicity, numerous septa, and high vasculature at Doppler examination	Low echogenicity intratesticular lobulated mass, with heterogeneous echotexture, low echogenicity, measuring 32 × 31 × 23 mm, with slight vasculature at Doppler examination	Rearranged aspect secondary to surgery Volume 12 mL

- Right testis: Normal in size and echotexture, without a focal lesion; normal epididymides; no varicocele; normal vasculature at Doppler examination.
- Left testis: a left hypoechoic polylobulated intratesticular mass measuring 30 × 16 mm, including numerous septa, and showing high vasculature at Doppler examination.

At that time, ofloxacine 200 mg twice per day during 15 days was prescribed. A spontaneaous pregnancy occurred in 2009 and a boy was born in 2010 (3800 g). Between 2009–2014 the patient had no follow-up for this mass.

In 2014 the patient consulted a doctor in our clinic as he wished to have another child. Semen analyses showed strict azoospermia with normal volume (Table 1).

Physical examination showed a normal andrological development, testes were present in the scrotum, a slightly indurated mass could be felt in the upper pole of the left testis. Right testis was hypotrophic, seminal conducts were felt. There was neither gynecomastia nor any sign of hypercortisolism.

Scrotal ultrasonography found (Table 2) a hypotrophic right testicle (8.5 mL), but a hypoechoic lobulated mass was visible in the left testicle with heterogeneous echotexture, measuring 32 × 31 × 23 mm, with slight vasculature at Doppler examination.

Serum analyses are described in Table 3. Total testosterone was measured by RIA using the Coat-A-Count Kit (Siemens Diagnostics, Inc., Los Angeles, CA, USA). The assay’s limit of quantification was 0.14 nmol/L (0.04 ng/mL) and the interassay CV was between 5.9 and 11.0 % for a range of concentrations between 0.76 and 13 ng/mL. The FSH, LH, and estradiol were measured using chemiluminescent, two-site immunoassays on a multiparameter analyzer (Architect; Abbott Laboratories). Delta4 androstenedione was measured in duplicate by radioimmunoassay using a kit provided by Beckman Coulter Immunotech. The percentage of testosterone cross-reactivity was 0.5 % with androstenedione and 0.02 % with estradiol.

**Table 3:** Hormonal results in 2014

	Normal range	Patient
LH IU/L	0.60–12	<0.1
Total testosterone ng / mL	2.30–6.7	4.61
Testosterone/SBP	28–78	46.19
Free testosterone pmol/L	188-444	311.04
FSH IU/L	1.2.–7.8	0.2
Inhibin B pg/mL	92–316	54
Delta4 androstenedione ng/mL	0.4–1.5	10.40
17 OH progesterone ng/mL	0.5–2.5	1.07
Dehydroepiandrosterone Sulfate μmol/L	1.7–12.8	6.0
Estradiol pg/mL	9–62	26
LDH IU/L	135–225	214
Human CG IU/L	<2.5	< 2
Alfa foeto protein μg/L	< 10	1
Cortisol (8 AM) μg / 100 mL	9–22	9.8
ACTH pg/mL	<46	41

*IU* international unit

*ng/mL* nanogram/milliliter

*pmol/L* picomole/liter

*IU/L* international unit/liter

*pg/mL* picogram/milliliter

*μmol/L* micromole/liter

*μg/L* microgram/liter

μg / 100 mL : microgram/100 milliliter

A marked increase in androstenedione levels and suppressed gonadotropin levels were found which are likely to contribute to the failure of spermatogenesis. Normal Dehydroepiandrosterone Sulfate argues against adrenal hyperandrogenism.

Abdomen and Thorax Computed Tomography (CT) imaging were normal (no adrenal gland abnormality and no secondary lesions).

The committee on tumoral diseases agreed on the decision to perform a testis-sparing surgery with extemporaneous histological examination. We decided to perform a testicular sperm extraction (TESE) at the same time. TESE allowed spermatozoa extraction with freezing.

**Fig. 1:**
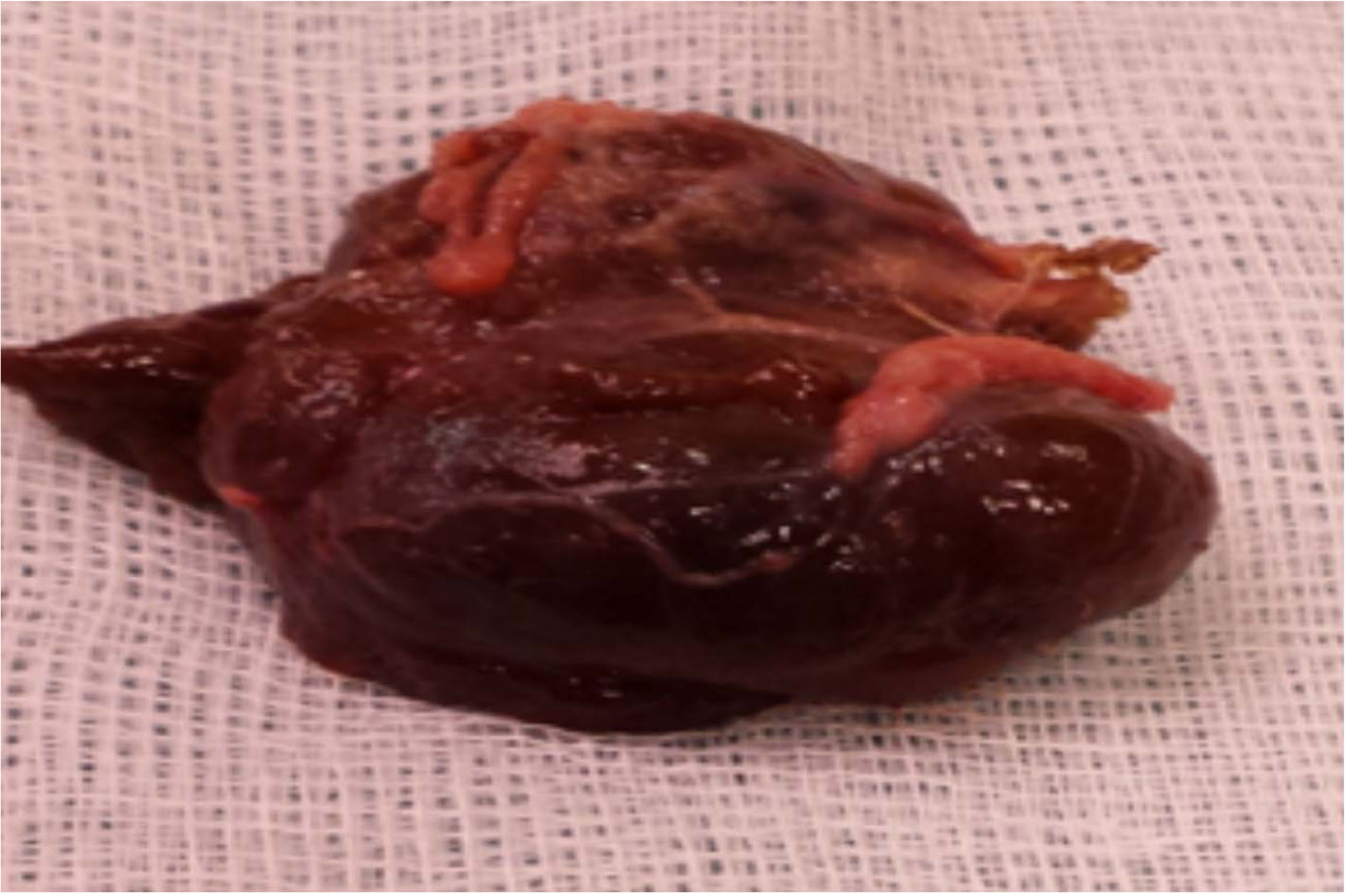
Surgical resection of the tumor. Tumor of 35 × 30 × 17 mm

Surgical resection of the tumor (Fig. 1) allowed a tumor of 35 × 30 × 17 mm to be removed.

**Fig. 2:**
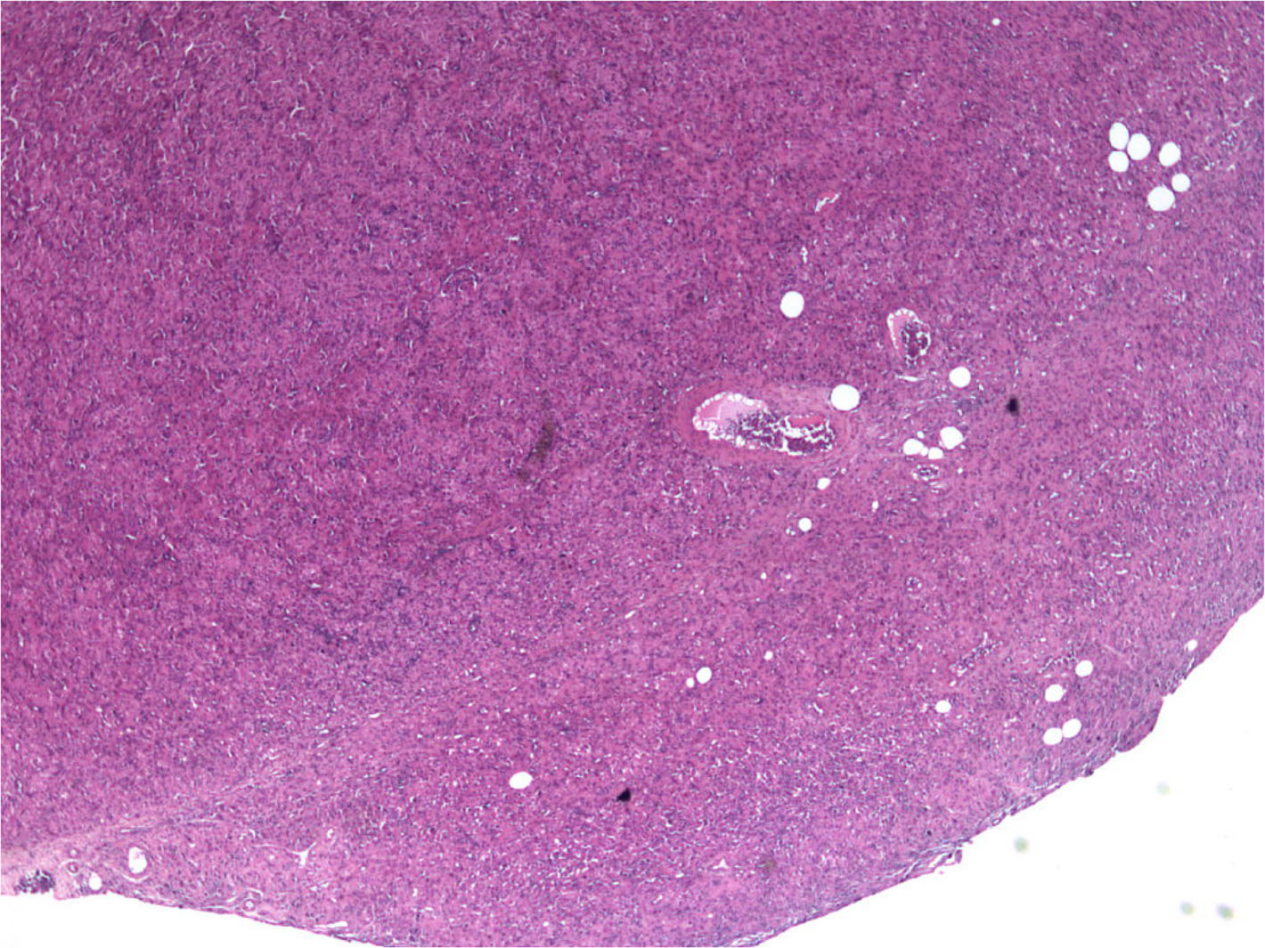
Photo of the tumor at low magnification (X50). Nodular and well limited tumor composed of sheets of eosinophilic cells with Hematoxylin Eosin and Saffron (HES) stain

**Fig. 3:**
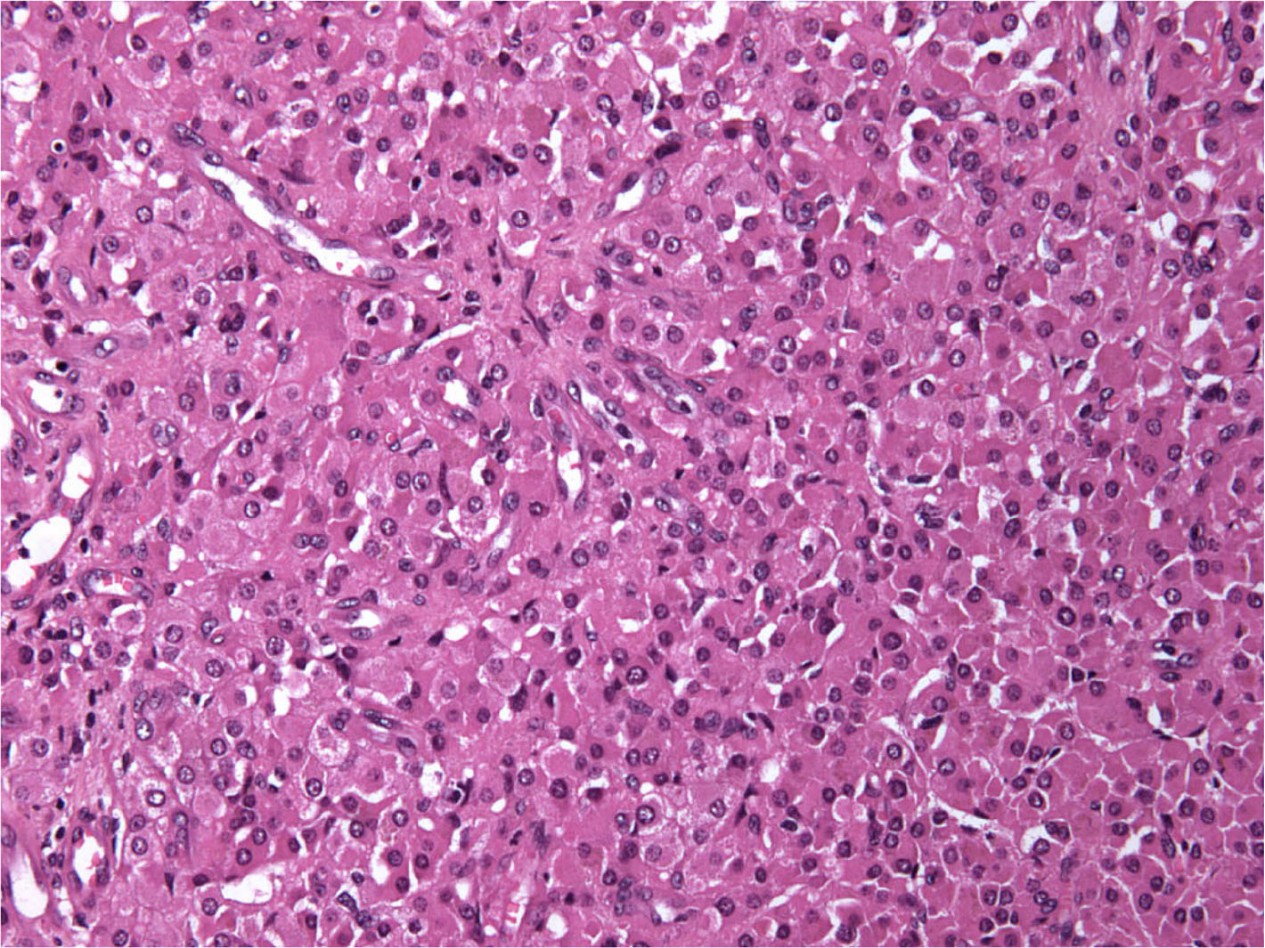
Photo of the tumor at high magnification (X200). Tumor cells are large with an abundant eosinophilic cytoplasm and round regular nuclei with small nucleoli, according with Leydig cells. Hematoxylin Eosin and Saffron (HES) stain

**Fig. 4:**
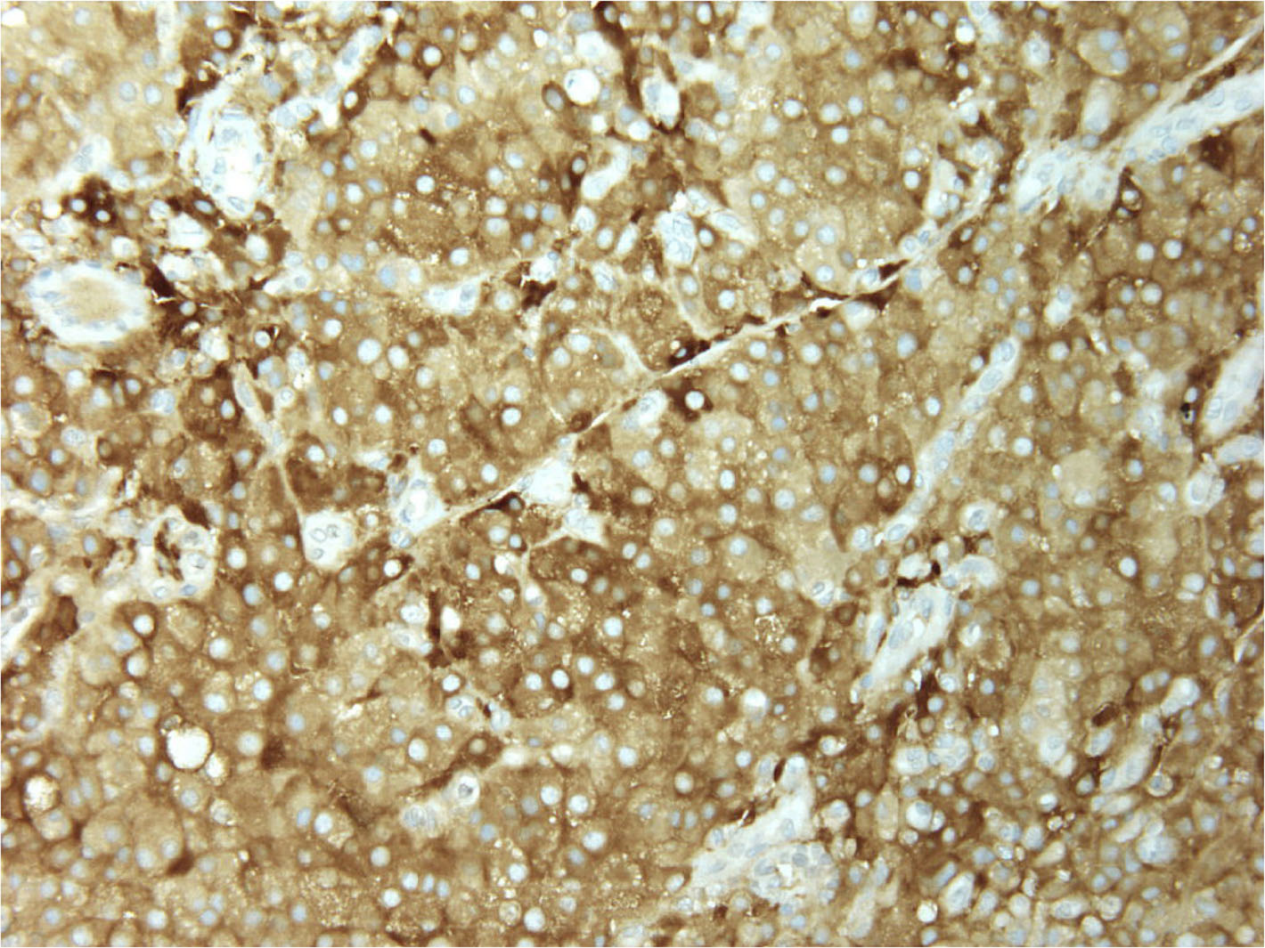
Photo of the tumor at high magnification (X400). Tumors cells were diffusely stained with antibody to inhibin A(immunoperoxydase). All tumor cells present a diffuse and strong cytoplasmic staining

Immunostaining was performed on an automated immunostainer (Benchmark, Ventana, France) with antibodies to Inhibin A (Ventana, prediluted, pretreatment: EDTA pH 7.8 for 60 min) and to SALL4 (Sigma, Dilution: 1/1500, pretreatment: EDTA pH 7.8 for 60 min). All tumor cells were diffusely and strongly stained with antibody to Inhibin A corresponding to Leydig cell tumor and were completely negative to antibodies to SALL4 showing that there was no germ cell contingent (Figs. 2, 3 and 4).

**Fig. 5:**
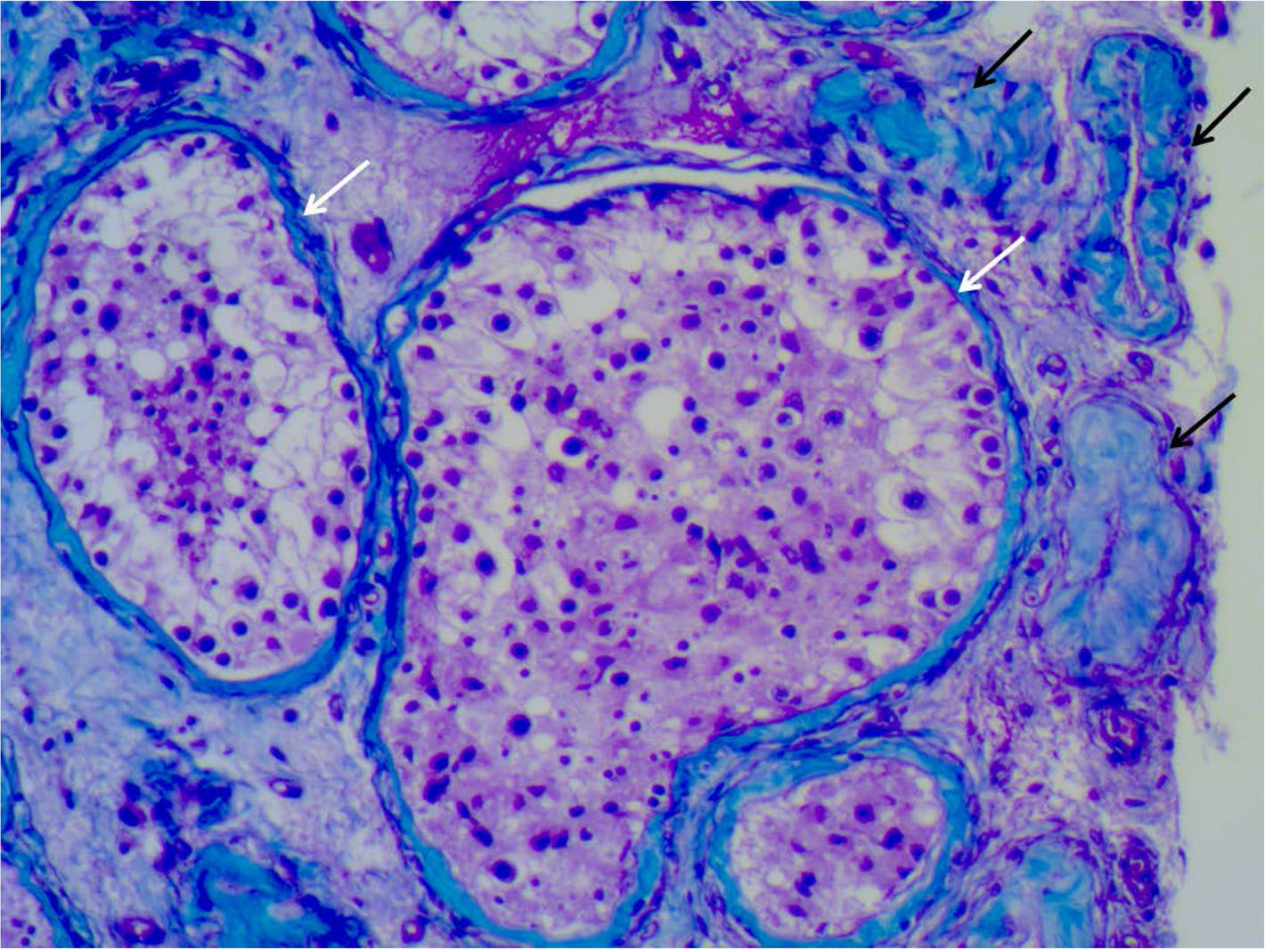
Photo of the testicular biopsy at high magnification (X100). Histopathological micrograph with hematoxylin-eosin-green FCF stain. The testicular biopsy consists of tubules with hypospermatogenesis (white arrows) mixed with aplasia (black arrows)

The testicular histology found hypospermatogenesis and germ-cell aplasia with interstitial fibrosis (Fig. 5).

Surgical resection of the tumor (Fig. 1) resulted in normalization of gonadotropins and fall in serum delta4 androstenedione to subnormal levels in the postoperative period confirming that the tumor was secreting delta4 androstenedione (Table 4).

**Table 4:** Selected hormonal values before and after surgery in 2014

	Normal range	1 month before surgery	1 day post-operative	2 months post-operative
LH (IU/L)	0.6–12.0	0.1	<0.1	1.6
Total Testosterone^a^ (ng/mL)	2.3–6.7	4.61	0.37	2.28
SBP (nmol/L)	12.5–42.2	34.6	ND	30.7
Delta4 androstenedione (ng/mL)	0.4–1.5	10.4	1.08	0.43
FSH (IU/L)	1.2–7.8	0.2	0.2	2.7
Inhibin B (pg/mL)	92–316	54	ND	82

*IU* international unit

*ng/mL* nanogram/ milliliter

*nmol/L* nanomole/ milliliter

*pg/mL* pictogram/ milliliter

*ND* not done

^a^To convert serum testosterone values from ng/mL into system international (SI) units (nmol/L), multiply by 3.47

**Fig. 6:**
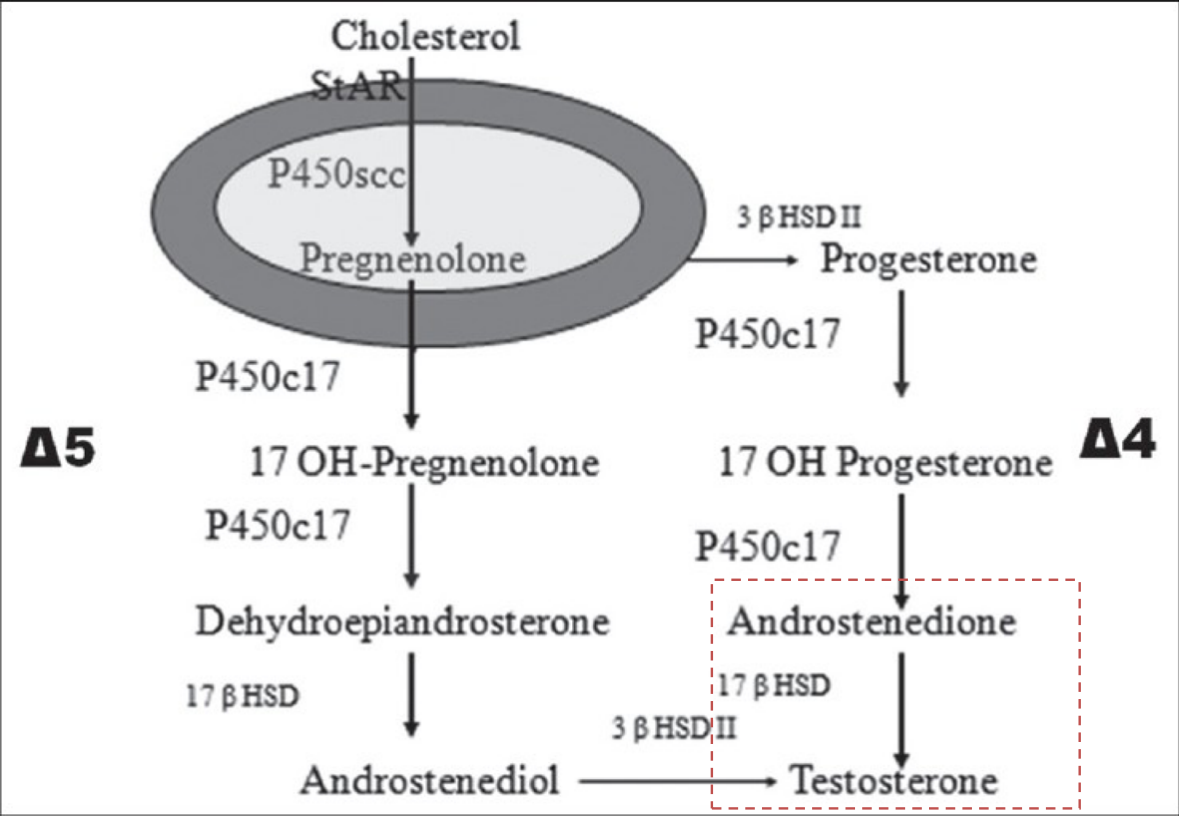
Testicular Steroidogenesis [21]

We hypothesized that high delta4 androstenedione resulted in intra tumoral 17 β-HSD overtaken by a delta4 androstenedione excess or that 17 betaHSD activity in the tumor was different from that of normal Leydig cells (Fig. 6).

Scrotal ultrasonography 3 months after surgery described a normal right testis which had increased in size (volume 15 mL) and a left testis with a rearranged aspect secondary to surgery (Table 2).

Three months after surgery semen analyses showed a complete recovery of spermatogenesis (Table 1). A spontaneous pregnancy occurred, 3 months after surgery and a girl was born in 2015 (3450 g).

## Discussion

To the authors’ knowledge only one case of androstenedione secreting testicular Leydig cell tumors associated with azoospermia has been reported previously [2]. In that case, serum levels of testosterone, dihydrotestosterone, 5α-androstane-3α,17βdiol and oestradiol were normal and oestrone was moderately increased. In contrast androstenedione was extremely elevated. Testosterone levels in the spermatic vein were decreased indicating a partial deficiency of 17β hydroxysteroid dehydrogenase in the tumoral tissue. Twenty-eight months after surgery, all sex steroids including androstenedione were normal.

In the present case, total suppression of LH levels and almost complete suppression of FSH levels were associated with azoospermia, despite intratesticular testosterone secretion by the tumor, and azoospermia proved to be fully reversible within less than three months after tumor removal and normalization of hormone levels, which shows that normal secretion of gonadotropins, plays a major role in maintaining spermatogenesis. This appears to be in agreement with other (rare) reports of patients with Leydig cell tumors and reversible azoospermia, who did not suffer from other testicular disorders. Interestingly, in these previous reports, the patients were also found to have testosterone secreting Leydig cell tumors with undetectable [15] or markedly reduced [12] gonadotropin levels, which suggests that intratesticular secretion of testosterone by the tumor is not sufficient to prevent azoospermia in spite of marked suppression of LH and FSH secretion. The present paper shows that even with collapsed gonadotropin levels TESE allowed extraction of spermatozoa.

## Conclusions

Hormone secreting interstitial cell tumors are rare and have variable clinical presentations. In the case presented in this paper, the diagnosis was made in a context of azoospermia. Few months after tumorectomy, sex steroid levels and spermatogenesis returned to normal and a spontaneous pregnancy occurred.

## Abbreviations

17 β-HSD: 17Beta hydroxysteroid dehydrogenase
CT: Computed tomography
FSH: Follicle stimulating hormone
LH: Luteinizing hormone
TESE: Testicular sperm extraction
